# Association of long-term alcohol consumption with DSA-defined concurrent intracranial and extracranial steno-occlusive involvement in acute ischemic stroke

**DOI:** 10.3389/fneur.2026.1881608

**Published:** 2026-08-25

**Authors:** Lei Jiang, Wenjiang Wu, Linghui Kong, Jiangyun Xiong, Xing Zhang, Weili Wang, Xiaojing Guo

**Affiliations:** 1 Department of Neurology, Huishui County People’s Hospital, Huishui, Guizhou, China; 2 Department of Neurology, The Affiliated Suzhou Hospital of Nanjing Medical University, Suzhou, China

**Keywords:** acute ischemic stroke, digital subtraction angiography, extracranial atherosclerosis, intracranial atherosclerosis, long-term alcohol consumption, steno-occlusive involvement

## Abstract

**Background:**

Intracranial and extracranial arteries differ in vascular wall structure and susceptibility to atherosclerosis. However, some patients with acute ischemic stroke have steno-occlusive lesions in both arterial compartments. The factors associated with this cross-compartment distribution remain unclear. We examined whether long-term alcohol consumption was associated with digital subtraction angiography (DSA)-defined concurrent intracranial and extracranial involvement.

**Methods:**

This retrospective study included patients with acute ischemic stroke who presented within 7 days of symptom onset and underwent cervicocerebral DSA at Huishui People’s Hospital, Guizhou, China, from January 2024 to March 2026. Long-term alcohol consumption was defined as drinking on at least 3 days per week for at least 5 years before the index stroke. Concurrent involvement was defined as at least one intracranial and at least one extracranial atherosclerotic steno-occlusive lesion in the same patient. The main multivariable logistic regression model included long-term alcohol consumption, age, sex, hypertension, diabetes mellitus, and smoking.

**Results:**

Among 249 patients, 59 (23.7%) had long-term alcohol consumption and 126 (50.6%) had concurrent involvement. The prevalence of long-term alcohol consumption was 11.8% in patients with isolated intracranial involvement, 19.1% in those with isolated extracranial involvement, and 32.5% in those with concurrent involvement (*p* = 0.003). After adjustment, long-term alcohol consumption remained associated with concurrent involvement (adjusted OR 2.57, 95% CI 1.21–5.50; *p* = 0.015). Compared with isolated intracranial involvement, long-term alcohol consumption was associated with higher odds of concurrent involvement (adjusted OR 2.73, 95% CI 1.09–6.79; *p* = 0.031). A similar but nonsignificant estimate was observed when isolated extracranial involvement was the comparator (adjusted OR 2.64, 95% CI 0.94–7.46; *p* = 0.066).

**Conclusion:**

In this DSA-selected cohort, long-term alcohol consumption remained associated with concurrent intracranial and extracranial steno-occlusive involvement after multivariable adjustment. This finding reflects a cross-compartment anatomical distribution pattern rather than overall atherosclerotic burden or stenosis severity. Given the retrospective single-center design, the findings should be interpreted cautiously and confirmed in larger prospective multicenter studies.

## 1 Introduction

Atherosclerotic steno-occlusive disease is an important vascular substrate of ischemic stroke. It is particularly relevant in Asian populations, in which intracranial atherosclerotic disease is common (1). Intracranial and extracranial arteries form a continuous cervicocerebral circulation, but they are not structurally identical vascular compartments. Compared with extracranial arteries, intracranial arteries generally have a thinner media and adventitia, fewer medial elastic fibers, a more prominent internal elastic lamina, an absent or poorly developed external elastic lamina, and sparse or absent vasa vasorum (2, 3). These regional differences may partly reflect developmental heterogeneity in vascular mural cell origin. Lineage-tracing studies have shown that cephalic neural crest cells contribute pericytes and vascular smooth muscle cells to vessels of the face and forebrain (4). These structural and developmental differences may contribute to distinct patterns of susceptibility to atherosclerosis.

Despite these differences, some patients have steno-occlusive lesions in both the intracranial and extracranial arterial compartments. Previous imaging studies have documented the coexistence of intracranial and extracranial atherosclerotic disease. They have also reported associations between this coexistence and ipsilateral cerebral infarction or recurrent vascular events (5–8). Concurrent involvement may therefore be viewed as a cross-compartment anatomical distribution pattern. This pattern describes disease distribution across two vascular compartments rather than total atherosclerotic burden or stenosis severity. However, the clinical and lifestyle factors associated with this pattern remain unclear.

Alcohol use has been associated with ischemic stroke risk, although the direction and magnitude of this association vary according to drinking pattern (9). However, whether long-term alcohol consumption is related to the distribution of steno-occlusive disease across both intracranial and extracranial arterial compartments remains unclear. Comprehensive cervicocerebral digital subtraction angiography (DSA) allows both arterial compartments to be assessed within a single examination (10). We used a DSA-selected cohort of patients with acute ischemic stroke. We examined the association between long-term alcohol consumption and concurrent intracranial and extracranial steno-occlusive involvement.

## 2 Methods

### 2.1 Study population and ethics

This retrospective single-center study screened consecutive patients with acute ischemic stroke who were admitted to the stroke unit of Huishui People’s Hospital, Guizhou Province, China, between January 2024 and March 2026. Clinical and imaging data were generated during routine diagnosis and treatment. The study protocol was approved by the institutional ethics committee on April 12, 2026 (approval number: HY2026-01). Approval was obtained before chart review, supplementary telephone data collection, database construction, and statistical analysis. The requirement for written informed consent was waived for the retrospective review. When alcohol-related information in the medical records was incomplete, supplementary data were collected by telephone after ethics approval. Verbal informed consent was obtained before each interview. All data were analyzed in de-identified form. The study was conducted in accordance with the Declaration of Helsinki.

Patients were eligible if they: (1) had acute ischemic stroke; (2) presented within 7 days of symptom onset; and (3) underwent cervicocerebral digital subtraction angiography during the index hospitalization. DSA was performed either to further evaluate vascular abnormalities identified during the inpatient diagnostic workup or as part of endovascular treatment, including mechanical thrombectomy.

Patients were excluded if they: (1) were functionally dependent before the index stroke; (2) had severe hepatic or renal dysfunction; (3) had severe infection; or (4) had missing data on long-term alcohol consumption, the primary outcome, or any covariate included in the main multivariable model. The final analytic cohort therefore comprised a DSA-selected subset of hospitalized patients with acute ischemic stroke.

### 2.2 Data sources and covariates

Demographic, clinical, laboratory, alcohol-related, and angiographic data were extracted from the hospital database and electronic medical records. Variables collected for the present analysis included age, sex, hypertension, diabetes mellitus, smoking status, hemoglobin, low-density lipoprotein cholesterol, random blood glucose, homocysteine, NIHSS score at admission, mRS score at admission, alcohol-related variables, and DSA findings. Stroke severity at admission was assessed using the NIHSS (11), and functional status at admission was assessed using the mRS (12).

### 2.3 Angiographic assessment and primary outcome

All patients underwent comprehensive cervicocerebral DSA during the index hospitalization. Angiographic coverage was sufficient to evaluate the bilateral cervical carotid and vertebral arteries, proximal subclavian arteries, bilateral intracranial anterior circulations, and vertebrobasilar circulation (10, 13). Angiographic findings were extracted directly from the final clinical DSA reports. These reports reflected the consensus interpretation of the neurointerventional team performing the procedure. At the time of angiographic interpretation, the neurointerventional team was unaware of the patients’ alcohol-consumption status.

For the purposes of the present study, the evaluated arteries were classified into extracranial and intracranial arterial compartments. The extracranial arterial compartment included the bilateral common carotid arteries, the extracranial segments of the bilateral internal carotid arteries, the bilateral external carotid arteries, the V1–V3 segments of the bilateral vertebral arteries, and the proximal segments of the bilateral subclavian arteries (13). The intracranial arterial compartment included the intracranial segments of the bilateral internal carotid arteries, the V4 segments of the bilateral vertebral arteries, the basilar artery, the M1–M3 segments of the middle cerebral arteries, the A1–A3 segments of the anterior cerebral arteries, and the P1–P3 segments of the posterior cerebral arteries. For study classification purposes, the internal carotid artery was divided into extracranial and intracranial segments at its entry into the carotid canal (14). The V1–V3 segments of the vertebral artery were classified as extracranial, whereas the V4 segment was classified as intracranial. Arterial branches distal to the M3, A3, and P3 segments were not included in the present analysis.

**Figure 1:**
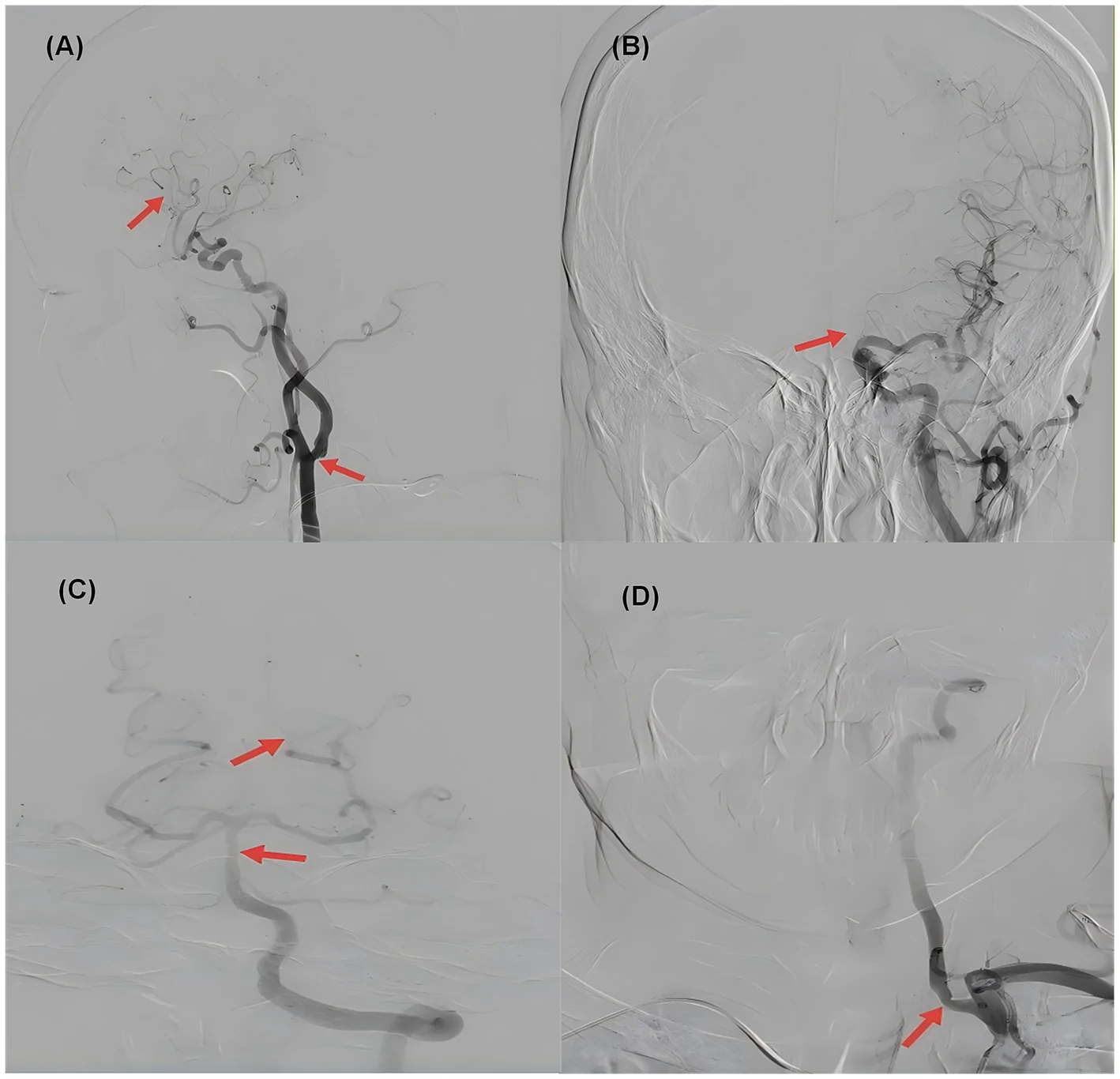
Representative digital subtraction angiography images illustrating concurrent involvement of the extracranial and intracranial arterial compartments. **(A,B)** Representative anterior-circulation case showing an extracranial stenotic lesion at the origin of the internal carotid artery and an intracranial occlusion of the anterior cerebral artery. **(C,D)** Representative posterior-circulation case showing an extracranial stenotic lesion at the origin of the vertebral artery and intracranial lesions consisting of mild basilar artery stenosis and posterior cerebral artery occlusion. Red arrows indicate the sites of steno-occlusive lesions.

The primary outcome was DSA-defined concurrent intracranial and extracranial steno-occlusive involvement. Concurrent involvement was defined as the presence of at least one angiographically documented steno-occlusive lesion in each arterial compartment. Representative angiographic examples of concurrent involvement are shown in Figure 1. Only lesions considered atherosclerotic in origin were included. Based on the stenosis severity documented in the final clinical DSA reports, qualifying lesions were categorized as mild (<50% luminal narrowing), moderate (50–69%), severe (70–99%), or occlusion (100%). These categories were consistent with commonly used angiographic severity classifications for extracranial and intracranial arterial stenosis (15, 16). Patients with qualifying steno-occlusive lesions in only one arterial compartment were classified as not having the primary outcome.

### 2.4 Alcohol exposure assessment

The primary exposure was long-term alcohol consumption. Alcohol history was obtained from the medical records during the index hospitalization. When the information in the medical records was incomplete, missing information was supplemented by a telephone interview conducted after ethics approval. Long-term alcohol consumption was defined *a priori* for the present study as alcohol intake on at least 3 days per week for at least 5 years before the index stroke. This operational definition was informed by previous studies evaluating habitual, sustained, or cumulative alcohol exposure over multiple years and its association with health outcomes, including stroke risk (17–20). Patients who did not meet this definition were classified in the no-long-term-drinking group. Drinking duration was recorded as the reported number of drinking years before the index stroke.

To further characterize the exposure profile of the long-term alcohol consumption group, a descriptive analysis of drinking intensity was performed. Alcohol by volume (ABV), drinking volume per occasion, and weekly drinking frequency were used to estimate ethanol intake. ABV was expressed in decimal form. Pure alcohol intake per occasion was calculated as beverage volume (mL) × ABV × 0.789 g/mL, where 0.789 g/mL represents the density of ethanol at approximately 20 °C (21). Weekly pure alcohol intake was calculated by multiplying pure alcohol intake per occasion by weekly drinking frequency. One US standard drink was defined as 14 g of pure alcohol (22).

For descriptive purposes, three threshold-based indicators were also applied. The weekly heavy-drinking indicator was defined as at least 15 US standard drinks per week for men and at least 8 US standard drinks per week for women. The sex-specific 5+/4 + −drink per-occasion indicator was defined as at least 5 US standard drinks per occasion for men and at least 4 US standard drinks per occasion for women (23). The World Health Organization 60-g-per-occasion threshold was defined as the consumption of at least 60 g of pure alcohol on a single occasion (24).

### 2.5 Statistical analysis

Continuous variables were summarized as medians and interquartile ranges, and categorical variables were summarized as numbers and percentages. Between-group comparisons were performed using the Mann–Whitney U test for continuous variables and the chi-square test or Fisher’s exact test for categorical variables, as appropriate. The prevalence of long-term alcohol consumption across isolated intracranial, isolated extracranial, and concurrent intracranial and extracranial involvement patterns was compared using the chi-square test. Wilson 95% confidence intervals were calculated for the observed proportions.

Univariable logistic regression was first used to examine the associations of long-term alcohol consumption, age, sex, hypertension, diabetes mellitus, and smoking with DSA-defined concurrent intracranial and extracranial steno-occlusive involvement. All six variables were then entered simultaneously into the main multivariable logistic regression model. Long-term alcohol consumption was the exposure of primary interest. Age, sex, hypertension, diabetes mellitus, and smoking were included as clinically relevant adjustment covariates.

Secondary and sensitivity analyses were also performed. First, concurrent intracranial and extracranial involvement was compared separately with isolated intracranial involvement and isolated extracranial involvement using multivariable logistic regression. The other isolated group was excluded from each comparison. These models were adjusted for age, sex, hypertension, diabetes mellitus, and smoking.

Second, the primary association was examined across sequentially adjusted models using a common complete-case sample. Model 1 included age, sex, hypertension, and diabetes mellitus. Model 2 additionally included smoking. Model 3 additionally included LDL-C, prior statin use, and previous stroke. Model 4 additionally included coronary artery disease.

Third, exploratory compartment-specific analyses evaluated intracranial and extracranial stenosis separately according to the highest stenosis grade within each arterial compartment. Two severity thresholds were examined: ≥50% stenosis and ≥70% stenosis or occlusion. These models were adjusted for age, sex, hypertension, diabetes mellitus, and smoking.

Exploratory analyses examined the association between drinking duration and the primary outcome among patients with long-term alcohol consumption. Drinking duration was analyzed both as a continuous variable per 10-year increase and in quartiles using empirical cut points of 20, 30, and 40 years. Univariable logistic regression was used for these analyses. A linear trend test was performed by treating quartile order as an ordinal variable, and the overall quartile effect was evaluated using a likelihood-ratio test. Odds ratios and 95% confidence intervals were reported.

An analysis-specific complete-case approach was used, and no missing-data imputation was performed. All statistical tests were two-sided, and *p* < 0.05 was considered statistically significant. Statistical analyses were performed using Python version 3.10 with the pandas, SciPy, and statsmodels libraries.

## 3 Results

### 3.1 Study population and baseline characteristics

A total of 249 patients with acute ischemic stroke who underwent cervicocerebral DSA during the index hospitalization were included in the final analysis. Of these, 59 met the definition of long-term alcohol consumption, and 190 were classified as not having long-term alcohol consumption. DSA-defined concurrent intracranial and extracranial steno-occlusive involvement was identified in 126 patients (50.6%).

Baseline characteristics according to long-term alcohol consumption status are summarized in Table 1. Compared with patients without long-term alcohol consumption, those with long-term alcohol consumption were more frequently male (93.2% vs. 55.3%, *p* < 0.001) and had a higher prevalence of smoking (71.2% vs. 14.7%, *p* < 0.001). They also had higher hemoglobin levels (139.0 [130.3, 149.0] vs. 133.0 [122.3, 145.8] g/L, *p* = 0.025). Concurrent intracranial and extracranial steno-occlusive involvement was more frequent in the long-term alcohol consumption group than in the group without long-term alcohol consumption (69.5% vs. 44.7%, *p* = 0.001). No statistically significant between-group differences were observed in age, LDL-C, random blood glucose, homocysteine, baseline NIHSS score, mRS score at admission, hypertension, or diabetes mellitus.

**Table 1:** Baseline characteristics and DSA-defined angiographic involvement according to long-term alcohol consumption status.

Variable	Overall (*n* = 249)	Group without long-term alcohol consumption (*n* = 190)	Long-term alcohol consumption group (*n* = 59)	*p* value
Age, years	66 (59, 72)	66 (58, 72)	65 (60, 70)	0.828
Hemoglobin, g/L	135.0 (124.0, 147.3)	133.0 (122.3, 145.8)	139.0 (130.3, 149.0)	0.025
LDL-C, mmol/L	2.4 (1.9, 3.0)	2.4 (1.8, 3.1)	2.5 (1.9, 2.9)	0.896
Random blood glucose, mmol/L	6.5 (5.6, 8.6)	6.5 (5.6, 8.5)	6.8 (5.6, 9.0)	0.665
Homocysteine, μmol/L	16.5 (13.3, 21.2)	16.3 (13.3, 20.7)	17.0 (13.4, 21.4)	0.517
Baseline NIHSS score	1 (0, 4)	1 (0, 5)	2 (0, 4)	0.585
mRS score at admission	1.0 (0.0, 3.0)	0.5 (0.0, 3.0)	1.0 (0.0, 2.5)	0.855
Male sex	160/249 (64.3%)	105/190 (55.3%)	55/59 (93.2%)	<0.001
Hypertension	212/249 (85.1%)	162/190 (85.3%)	50/59 (84.7%)	1.000
Diabetes mellitus	54/249 (21.7%)	40/190 (21.1%)	14/59 (23.7%)	0.718
Smoking	70/249 (28.1%)	28/190 (14.7%)	42/59 (71.2%)	<0.001
Concurrent intracranial and extracranial steno-occlusive involvement	126/249 (50.6%)	85/190 (44.7%)	41/59 (69.5%)	0.001

Values are presented as median (interquartile range) or *n*/*N* (%). *p* values were derived using the Mann–Whitney U test for continuous variables and the chi-square test or Fisher exact test for categorical variables, as appropriate. Long-term alcohol consumption was defined as alcohol intake on ≥3 days per week for ≥5 years before the index stroke. Concurrent intracranial and extracranial steno-occlusive involvement was defined as the presence, in the same patient, of at least one angiographically documented steno-occlusive lesion in the intracranial arterial compartment and at least one steno-occlusive lesion in the extracranial arterial compartment. Patients who did not meet the definition of long-term alcohol consumption constituted the comparison group. LDL-C data were missing for four participants; all other variables presented in this table were complete. LDL-C, low-density lipoprotein cholesterol; NIHSS, National Institutes of Health Stroke Scale; mRS, modified Rankin Scale; DSA, digital subtraction angiography.

### 3.2 Distribution of DSA-defined angiographic involvement patterns

Among the 249 patients, 76 had isolated intracranial involvement, 47 had isolated extracranial involvement, and 126 had concurrent intracranial and extracranial involvement. Representative DSA images of concurrent involvement of the two arterial compartments are shown in Figure 1.

**Figure 2:**
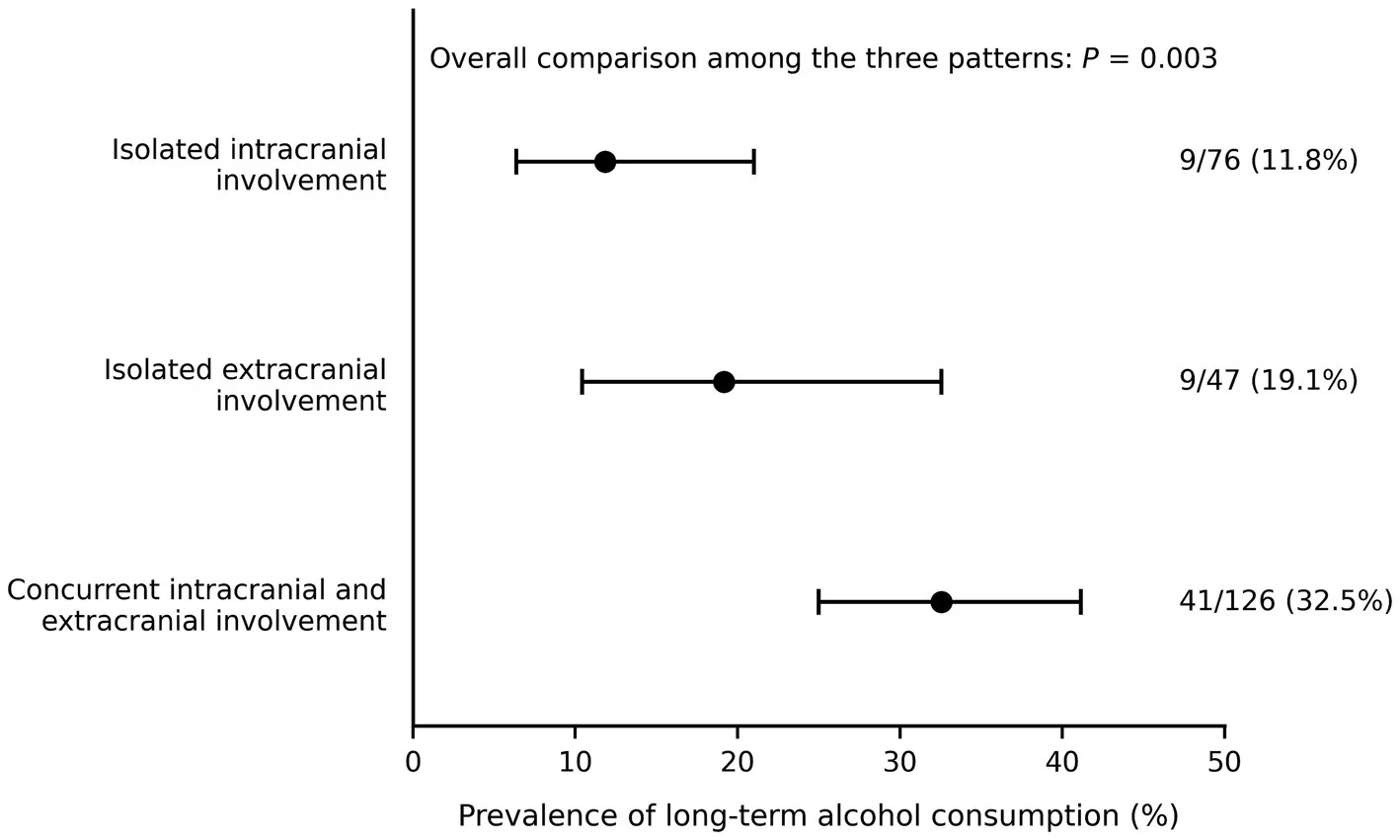
Prevalence of long-term alcohol consumption across DSA-defined angiographic involvement patterns. Points represent the observed prevalence of long-term alcohol consumption, and horizontal error bars represent Wilson 95% confidence intervals. The unadjusted overall difference among the three angiographic involvement patterns was assessed using the chi-square test (*p* = 0.003). DSA, digital subtraction angiography.

The prevalence of long-term alcohol consumption differed across the three angiographic involvement patterns. Long-term alcohol consumption was present in 9 of 76 patients with isolated intracranial involvement (11.8%), 9 of 47 patients with isolated extracranial involvement (19.1%), and 41 of 126 patients with concurrent intracranial and extracranial involvement (32.5%). The overall difference among the three groups was statistically significant (*p* = 0.003; Figure 2).

### 3.3 Association between long-term alcohol consumption and concurrent intracranial and extracranial involvement

The univariable and multivariable logistic regression results are presented in Table 2. In univariable analyses, long-term alcohol consumption was associated with concurrent intracranial and extracranial steno-occlusive involvement (OR 2.81, 95% CI 1.51–5.25, *p* = 0.001). Male sex (OR 2.54, 95% CI 1.49–4.33, *p* < 0.001), hypertension (OR 2.80, 95% CI 1.32–5.96, *p* = 0.007), and smoking (OR 1.84, 95% CI 1.05–3.24, *p* = 0.034) were also associated with the outcome.

**Table 2:** Logistic regression analyses of concurrent intracranial and extracranial steno-occlusive involvement.

Variable	Unadjusted OR (95% CI)	*p* value	Adjusted OR (95% CI)	*p* value
Long-term alcohol consumption	2.81 (1.51–5.25)	0.001	2.57 (1.21–5.50)	0.015
Age, years	1.00 (0.98–1.02)	0.999	1.00 (0.97–1.03)	0.970
Male sex	2.54 (1.49–4.33)	<0.001	2.36 (1.26–4.44)	0.008
Hypertension	2.80 (1.32–5.96)	0.007	3.25 (1.46–7.27)	0.004
Diabetes mellitus	1.42 (0.77–2.61)	0.260	1.46 (0.77–2.77)	0.244
Smoking	1.84 (1.05–3.24)	0.034	0.73 (0.34–1.53)	0.398

The outcome was DSA-defined concurrent intracranial and extracranial steno-occlusive involvement. Unadjusted ORs were estimated using separate univariable logistic regression models. Adjusted ORs were obtained from a multivariable logistic regression model including long-term alcohol consumption, age, sex, hypertension, diabetes mellitus, and smoking. CI, confidence interval; DSA, digital subtraction angiography; OR, odds ratio.

After adjustment for age, sex, hypertension, diabetes mellitus, and smoking, long-term alcohol consumption remained significantly associated with concurrent intracranial and extracranial involvement (adjusted OR 2.57, 95% CI 1.21–5.50, *p* = 0.015). Male sex (adjusted OR 2.36, 95% CI 1.26–4.44, *p* = 0.008) and hypertension (adjusted OR 3.25, 95% CI 1.46–7.27, *p* = 0.004) also remained significantly associated with the outcome. Smoking was no longer statistically significant after adjustment (adjusted OR 0.73, 95% CI 0.34–1.53, *p* = 0.398).

### 3.4 Drinking-intensity characteristics among patients with long-term alcohol consumption

All 59 patients in the long-term alcohol consumption group had complete drinking-intensity data. Their drinking-intensity characteristics are summarized in Table 3. The median ABV was 52.0% (20.0–52.0%), the median beverage volume per occasion was 150 mL (100–250 mL), and the median drinking frequency was 7 times per week (6–7 times per week).

**Table 3:** Descriptive drinking-intensity profile of the long-term alcohol consumption group.

Measure	Long-term alcohol consumption group (*n* = 59)
Alcohol by volume (ABV), %	52.0 (20.0, 52.0)
Beverage volume per occasion, mL	150 (100, 250)
Drinking frequency, times/week	7 (6, 7)
Pure alcohol per occasion, g	41.0 (22.1, 61.5)
Pure alcohol per week, g	276.2 (153.9, 430.8)
US standard drinks per occasion (14 g/drink)	2.9 (1.6, 4.4)
US standard drinks per week (14 g/drink)	19.7 (11.0, 30.8)
Met the NIAAA weekly heavy-drinking threshold, *n* (%)	34/59 (57.6%)
Met the sex-specific ≥5/≥4-drink per-occasion threshold, *n* (%)	13/59 (22.0%)
Met the WHO 60-g per-occasion threshold, *n* (%)	20/59 (33.9%)

Values are presented as median (interquartile range) or *n*/*N* (%). All 59 patients in the long-term alcohol consumption group had complete drinking-intensity data. Pure alcohol intake was calculated as beverage volume (mL) × alcohol by volume × 0.789 g/mL. One US standard drink equals 14 g of pure alcohol. The NIAAA weekly heavy-drinking threshold was defined as ≥15 drinks/week for men and ≥8 drinks/week for women. The sex-specific per-occasion threshold was defined as ≥5 US standard drinks for men and ≥4 US standard drinks for women. The WHO 60-g per-occasion threshold was defined as ≥60 g of pure alcohol on one occasion. ABV, alcohol by volume; NIAAA, National Institute on Alcohol Abuse and Alcoholism; WHO, World Health Organization.

The median pure alcohol intake was 41.0 g per occasion (22.1–61.5 g) and 276.2 g per week (153.9–430.8 g). When expressed as US standard drinks, the median intake was 2.9 drinks per occasion (1.6–4.4 drinks) and 19.7 drinks per week (11.0–30.8 drinks). Overall, 34 patients (57.6%) met the NIAAA weekly heavy-drinking threshold, 13 (22.0%) met the sex-specific ≥5/≥4-drink per-occasion threshold, and 20 (33.9%) met the WHO 60-g per-occasion threshold.

### 3.5 Secondary, sensitivity, and exploratory analyses

The secondary and sensitivity analyses and the exploratory compartment-specific analyses are presented in Table 4. In analyses using alternative comparator groups, long-term alcohol consumption was associated with concurrent intracranial and extracranial involvement when the comparison was restricted to patients with isolated intracranial involvement (adjusted OR 2.73, 95% CI 1.09–6.79, *p* = 0.031). When concurrent involvement was compared with isolated extracranial involvement, the adjusted OR was of similar magnitude, but the association did not reach statistical significance (adjusted OR 2.64, 95% CI 0.94–7.46, *p* = 0.066).

**Table 4:** Secondary, sensitivity, and exploratory analyses of the association between long-term alcohol consumption and DSA-defined arterial involvement.

(A) Analyses using alternative comparator groups					
Comparison	*n*	Concurrent involvement, *n*	Comparator group, *n*	Adjusted OR (95% CI)	*p* value
Concurrent involvement vs. isolated intracranial involvement	202	126	76	2.73 (1.09–6.79)	0.031
Concurrent involvement vs. isolated extracranial involvement	173	126	47	2.64 (0.94–7.46)	0.066

(B) Sequential adjustment of the primary association				
Model	Adjustments	*n*	Adjusted OR (95% CI)	*p* value
Model 1	Age, sex, hypertension, and diabetes mellitus	245	2.20 (1.12–4.34)	0.022
Model 2	Model 1 plus smoking	245	2.50 (1.16–5.36)	0.019
Model 3	Model 2 plus LDL-C, prior statin use, and previous stroke	245	2.49 (1.15–5.40)	0.021
Model 4	Model 3 plus coronary artery disease	245	2.40 (1.10–5.23)	0.028

(C) Exploratory analyses of stenosis-severity thresholds within each arterial compartment				
Outcome	*n*	Outcome events, *n*	Adjusted OR (95% CI)	*p* value
Intracranial stenosis ≥50%	249	142	3.34 (1.52–7.36)	0.003
Intracranial stenosis ≥70% or occlusion	249	117	2.24 (1.08–4.64)	0.030
Extracranial stenosis ≥50%	249	124	1.58 (0.77–3.25)	0.216
Extracranial stenosis ≥70% or occlusion	249	91	1.18 (0.57–2.46)	0.650

ORs compare patients with and without long-term alcohol consumption. In Panel A, concurrent involvement was compared separately with isolated intracranial and isolated extracranial involvement; the other isolated group was excluded from each comparison. In Panel B, all models used the same complete-case sample of 245 patients because LDL-C data were missing for four patients. Model 1 adjusted for age, sex, hypertension, and diabetes mellitus; Model 2 additionally included smoking; Model 3 additionally included LDL-C, prior statin use, and previous stroke; and Model 4 additionally included coronary artery disease. The primary analysis of all 249 patients is shown in Table 2. In Panel C, each arterial compartment was analyzed according to its highest stenosis grade. Stenosis ≥50% included moderate or severe stenosis and occlusion; stenosis ≥70% or occlusion included severe stenosis and occlusion. These analyses were exploratory, and differences in statistical significance should not be interpreted as evidence of different associations between the two arterial compartments. Models in Panels A and C were adjusted for age, sex, hypertension, diabetes mellitus, and smoking. CI, confidence interval; DSA, digital subtraction angiography; LDL-C, low-density lipoprotein cholesterol; OR, odds ratio.

In the common complete-case sample of 245 patients, the association between long-term alcohol consumption and concurrent involvement remained statistically significant across all sequentially adjusted models. The adjusted ORs ranged from 2.20 to 2.50, and the *p* values ranged from 0.019 to 0.028.

In the exploratory compartment-specific analyses, long-term alcohol consumption was associated with intracranial stenosis ≥50% (adjusted OR 3.34, 95% CI 1.52–7.36, *p* = 0.003) and intracranial stenosis ≥70% or occlusion (adjusted OR 2.24, 95% CI 1.08–4.64, *p* = 0.030). No statistically significant association was observed for extracranial stenosis ≥50% (adjusted OR 1.58, 95% CI 0.77–3.25, *p* = 0.216) or extracranial stenosis ≥70% or occlusion (adjusted OR 1.18, 95% CI 0.57–2.46, *p* = 0.650).

### 3.6 Exploratory analyses of drinking duration

Among the 59 patients with long-term alcohol consumption, drinking duration was not associated with concurrent intracranial and extracranial involvement when analyzed as a continuous variable per 10-year increase (OR 0.90, 95% CI 0.58–1.41, *p* = 0.653).

When drinking duration was categorized into quartiles using cut points of 20, 30, and 40 years, neither the overall difference across quartiles nor the linear trend was statistically significant. The likelihood-ratio test for the overall quartile effect yielded *p* = 0.637. The ordinal trend analysis yielded an OR of 0.78 per quartile increase (95% CI 0.45–1.37, *p* = 0.390). These exploratory findings did not indicate a clear association between longer drinking duration and concurrent intracranial and extracranial involvement among patients with long-term alcohol consumption (Supplementary Table S1).

## 4 Discussion

The principal finding of this study was that long-term alcohol consumption remained associated with DSA-defined concurrent intracranial and extracranial steno-occlusive involvement after adjustment for age, sex, hypertension, diabetes mellitus, and smoking. Long-term alcohol consumption was most prevalent among patients with concurrent involvement. The direction of this association was generally consistent across the secondary and sensitivity analyses. However, drinking duration did not show a clear graded association with the outcome.

The vascular significance of this finding lies primarily in the anatomical distribution of the disease. Although the intracranial and extracranial arteries form a continuous cervicocerebral circulation, they differ in vascular-wall architecture. These differences include the organization of the media, adventitia, elastic laminae, and vasa vasorum and may contribute to distinct patterns of susceptibility to atherosclerotic disease (2, 25, 26). Concurrent involvement therefore represents steno-occlusive disease across two structurally different arterial compartments in the same patient. The observed association suggests that long-term alcohol consumption may be relevant to this cross-compartment distribution rather than only to disease within a single arterial bed. However, the present findings do not establish that alcohol affects the two arterial compartments through the same biological pathway. Concurrent involvement should therefore be interpreted as an anatomical distribution pattern rather than a measure of total lesion number, overall atherosclerotic burden, stenosis severity, or clinical prognosis.

Previous research on alcohol and cerebrovascular disease has mainly focused on overall stroke risk, ischemic stroke subtypes, cumulative alcohol exposure, or vascular disease within a single arterial territory (18, 27–29). The relationship between alcohol exposure and cerebrovascular disease may vary according to drinking quantity, frequency, duration, and pattern. It may also vary according to population characteristics and accompanying vascular risk factors (19, 20, 30, 31). Population-based studies have linked higher, sustained, or cumulative alcohol exposure to an increased risk of stroke (32). More directly related to the present study, Liu et al. reported an association between alcohol consumption and cerebrovascular stenosis in patients with acute ischemic stroke or transient ischemic attack (33). However, that study evaluated the presence and severity of carotid and cerebral arterial stenosis rather than the distribution of lesions across the intracranial and extracranial arterial compartments. The present study extends this evidence by focusing specifically on a DSA-defined cross-compartment anatomical pattern.

Unlike previous studies that primarily characterized alcohol exposure according to total alcohol intake, cumulative consumption, or heavy-drinking thresholds, the present study focused on long-term habitual alcohol consumption as a sustained lifestyle pattern (22, 34–36). This exposure can be identified through routine history taking and is therefore practical for clinical assessment. To our knowledge, previous studies have not specifically examined the association between long-term habitual alcohol consumption and concurrent involvement of the intracranial and extracranial arterial compartments. The present finding therefore represents a clinical morphological observation linking a sustained drinking pattern to steno-occlusive disease across two anatomically distinct cerebrovascular beds in the same patient. It concerns the cross-compartment distribution of vascular disease rather than simply stenosis severity or overall atherosclerotic burden.

Clinically, a history of long-term alcohol consumption may provide readily obtainable context when interpreting cerebrovascular lesion distribution after comprehensive vascular imaging. However, alcohol history alone should not be used as an indication for invasive DSA (37).

The secondary and sensitivity analyses provided additional context for the primary finding. Long-term alcohol consumption was associated with concurrent involvement when isolated intracranial involvement was used as the comparator. A similar effect estimate was observed when isolated extracranial involvement was used as the comparator, although this association was not statistically significant. The effect estimates also remained similar across the sequentially adjusted models. In the exploratory compartment-specific analyses, long-term alcohol consumption was associated with intracranial stenosis defined at both severity thresholds but not with the corresponding extracranial outcomes. However, the difference in statistical significance does not demonstrate that the associations differed between the two arterial compartments. These results should therefore be interpreted cautiously and regarded as exploratory (38–40).

Several limitations should be acknowledged. First, this retrospective, single-center study included a clinically selected subgroup of patients with acute ischemic stroke who underwent DSA. Because DSA was performed for further vascular evaluation or endovascular treatment, the cohort may have been enriched for clinically important large-artery abnormalities. The findings are therefore most applicable to patients undergoing clinically indicated DSA and may not be generalizable to the broader acute ischemic stroke population. The observational design also precludes causal inference. Second, alcohol exposure was based on medical records and supplementary telephone interviews and was susceptible to recall and classification bias. Comprehensive lifetime exposure information was unavailable (34, 35). Third, angiographic findings were derived from final clinical DSA reports without independent core-laboratory reassessment. The outcome reflected anatomical distribution rather than total lesion burden or prognosis. Fourth, the modest sample size, particularly in the long-term alcohol-consumption and isolated extracranial groups, limited the precision of the secondary and exploratory analyses. Residual confounding cannot be excluded.

## 5 Conclusion

In this DSA-selected cohort of patients with acute ischemic stroke, long-term alcohol consumption remained associated with concurrent intracranial and extracranial steno-occlusive involvement. This finding concerns a cross-compartment anatomical distribution pattern rather than total atherosclerotic burden or stenosis severity. Larger prospective multicenter studies with standardized vascular assessment and more comprehensive measurement of alcohol exposure are needed to confirm this association.

## Data Availability

The original contributions presented in the study are included in the article/Supplementary material, further inquiries can be directed to the corresponding author.
