# Supplementary material for: Association of long-term alcohol consumption with DSA-defined concurrent intracranial and extracranial steno-occlusive involvement in acute ischemic stroke
Source: Front Neurol. 2026 Aug 25;17:1881608. doi: 10.3389/fneur.2026.1881608 (PMC13549890; doi:10.3389/fneur.2026.1881608)
Supplement: Supplementary file 1 [file Table_1.DOCX]

**Supplementary Table S1. Exploratory analyses of drinking duration among patients with long-term alcohol consumption**

*Panel A. Continuous analysis*

| **Exposure** | **n** | **Unadjusted OR (95% CI)** | **P value** |
| --- | --- | --- | --- |
| Drinking duration, per 10-year increase | 59 | 0.90 (0.58-1.41) | 0.653 |

*Panel B. Duration-category distribution and global tests*

| **Analysis** | **Duration range** | **n** | **Events** | **Event rate** | **Result** |
| --- | --- | --- | --- | --- | --- |
| Q1 | ≤20 years | 17 | 12 | 70.6% | - |
| Q2 | >20 to 30 years | 19 | 15 | 78.9% | - |
| Q3 | >30 to 40 years | 16 | 10 | 62.5% | - |
| Q4 | >40 years | 7 | 4 | 57.1% | - |
| Linear trend | - | 59 | - | - | OR 0.78 (0.45-1.37); P=0.390 |
| Overall test | - | 59 | - | - | LR χ²=1.70; df=3; P=0.637 |

Note. The linear trend test treated duration-category order as an ordinal variable. The overall comparison was based on a likelihood-ratio test comparing the duration-category model with the intercept-only model.
